# Supplementary material for: Deficiency of a triterpene pathway results in humidity-sensitive genic male sterility in rice
Source: Nat Commun. 2018 Feb 9;9:604. doi: 10.1038/s41467-018-03048-8 (PMC5807508; doi:10.1038/s41467-018-03048-8)
Supplement: Supplementary file 1 — Supplementary Information [file 41467_2018_3048_MOESM1_ESM.pdf]

Supplementary Information

Deficiency of a triterpene pathway results in humidity-sensitive genic male sterility in rice

By Xue *et al.*

Supplementary Figures:

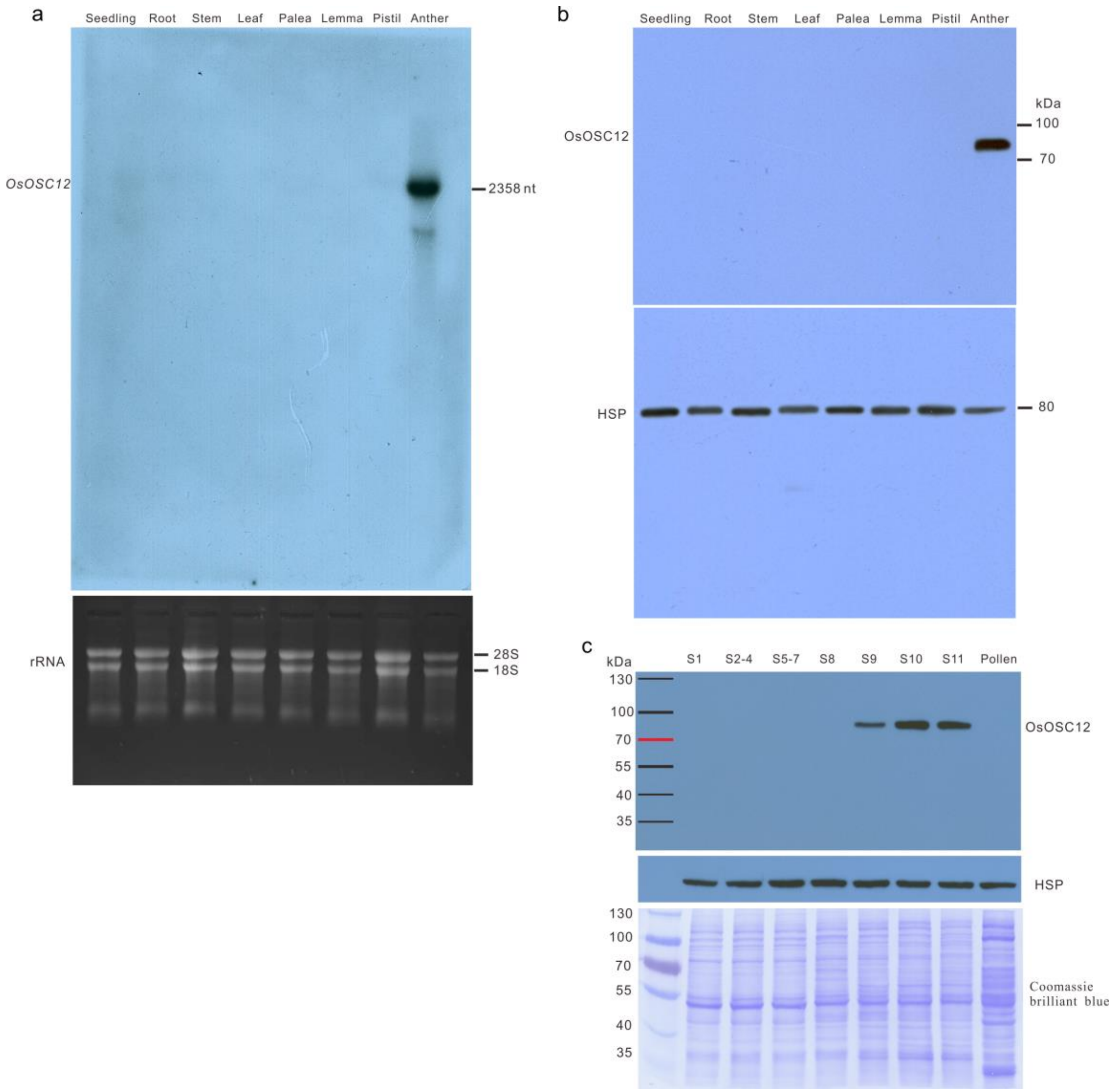

Supplementary Figure 1 | Transcription and expression of *OsOSC12*. (**a, b**) Tissue specificity as revealed by Northern and Western blotting. (**c**) Temporal profiles during anther development, as shown by Western blotting. S1 to S11 correspond to the stages of anther development as described in main text. The negative controls for Northern and Western blotting experiments were, respectively, 18S and 28S rRNA, and the rice heat shock protein Q69QQ6 and SDS-PAGE gel stained by coomassie brilliant blue.

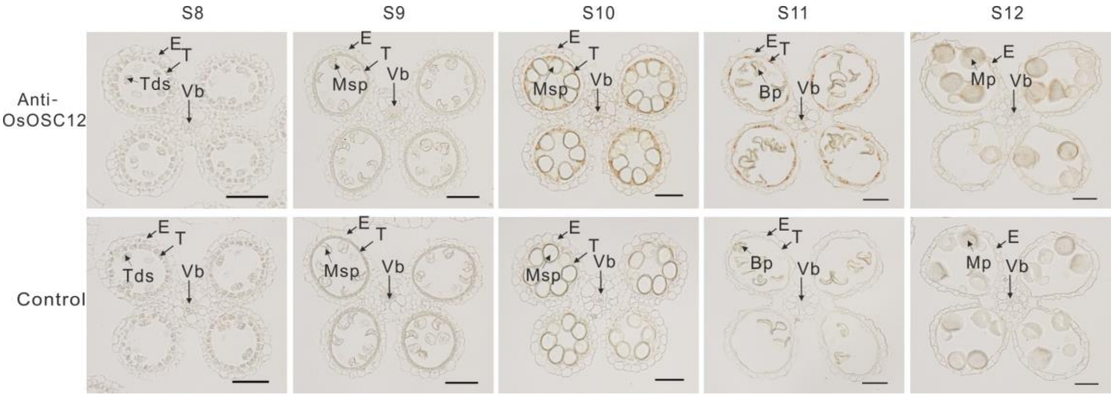

Supplementary Figure 2 | Expression pattern of OsOSC12 as revealed by immunolocalization. OsOSC12 accumulated from S9 in the tapetum and vascular bundle, and declined during S11 and S12 (the negative control contained only blocking buffer). Bp, bicellular pollen; E, epidermis; Mp, mature pollen; Msp, microspore; T, tapetum; Tds, tetrads; Vb, vascular bundle. Scale bar, 50 μm.

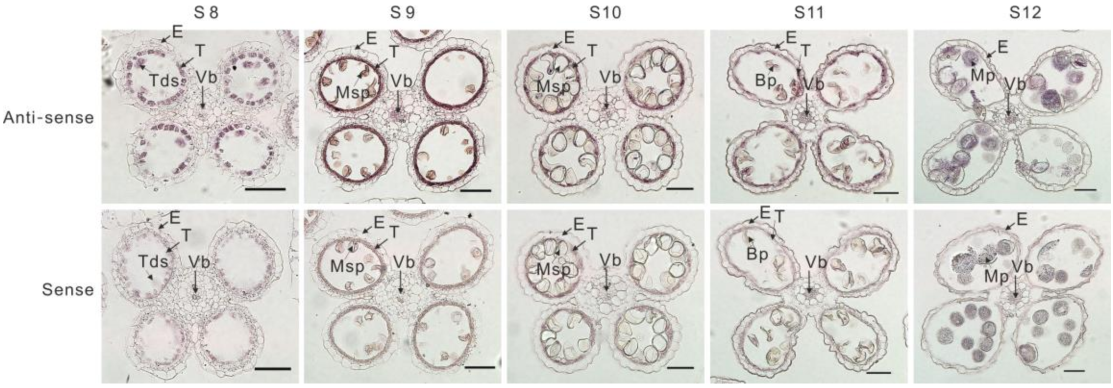

Supplementary Figure 3 | *In situ* mRNA analysis of *OsOSC12* transcript. A low abundance of transcript was present in the tapetum and tetrads at S8, after which the level rose until its decline during S10 and S11, falling below the level of detection by S12. The *OsOSC12* sense probe was used as the negative control. Bp, bicellular pollen; E, epidermis; Mp, mature pollen; Msp, microspore; T, tapetum; Tds, tetrads; Vb, vascular bundle. Scale bar, 50 µm.

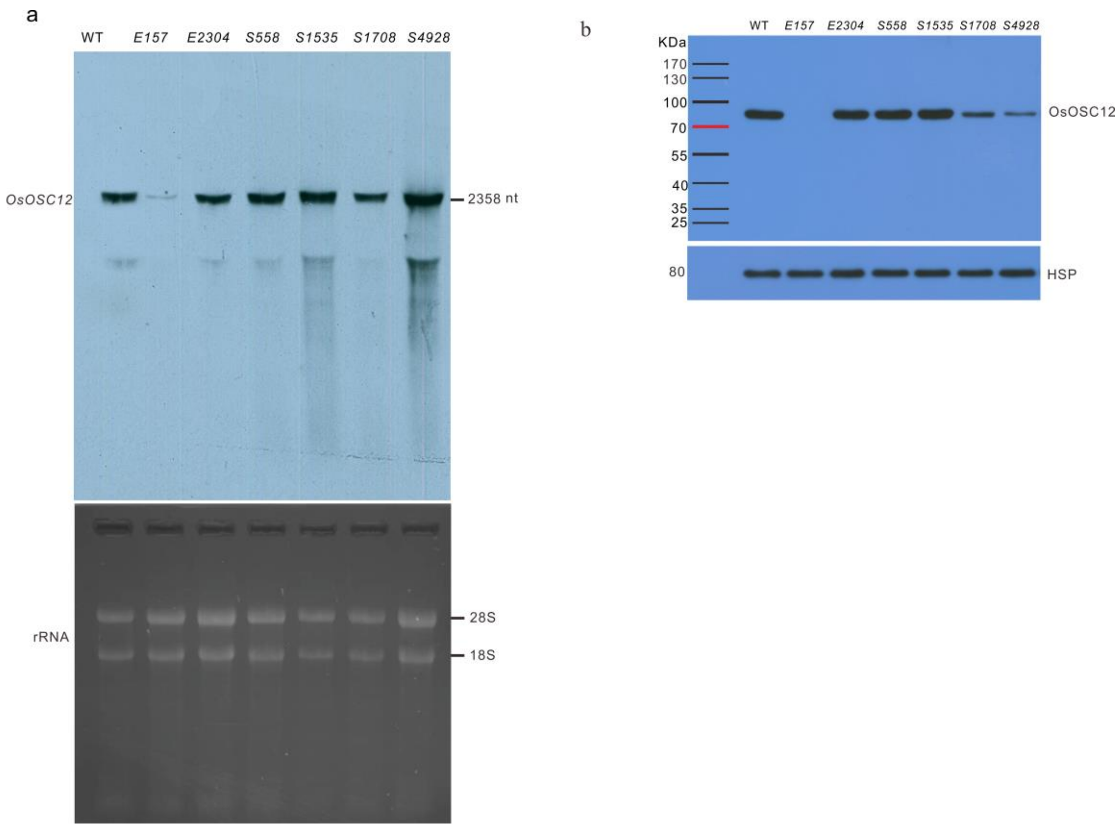

Supplementary Figure 4 | Transcription and expression of *OsOSC12* in mutants. (**a**) Northern blot analysis of *OsOSC12* transcription. (**b**) Western blot analysis of OsOSC12 expression. The negative controls for Northern and Western blotting experiments were, respectively, 18S and 28S rRNA, and the rice heat shot protein Q69QQ6.

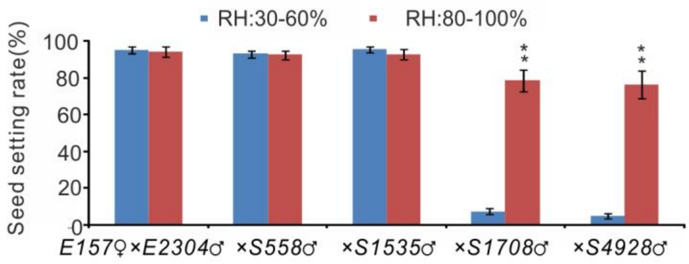

Supplementary Figure 5 | Seed setting of F_1 plants from crosses of *E157* with other mutants at the normal (30-60%) and high (80-100%) RH. The data are presented as means ± s.d., $n = 15$. $^{**}P < 0.01$, Student's t tests. Both the *E157* x *S1708* and *E157* x *S4928* F_1 hybrids were male sterile, while those derived from crosses of *E157* with each of the other three *OsOSC12* mutants were all male fertile.

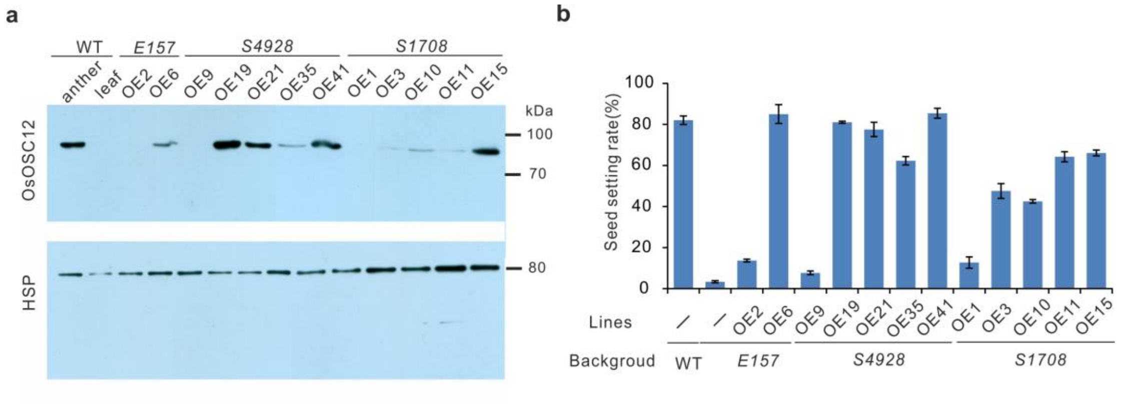

Supplementary Figure 6 | Complementary experiment of *OsOSC12*. (**a**) Western blotting shows the protein level of OsOSC12 (top-panel) in the WT and in the leave of twelve independent T_0 transgenic lines of *E157*，*S4928* and *S1708*. The rice heat shock protein Q69QQ6 is used as negative control (lower-panel) (**b**) Seed setting of the twelve T_0 lines of *E157*, *S4928* and *S1708* mutants at normal (30-60%) RH. The data are presented as means ± s.d., *n* = 6. The *OsOSC12* over-expression construct was driven by ubiquitin promoter.

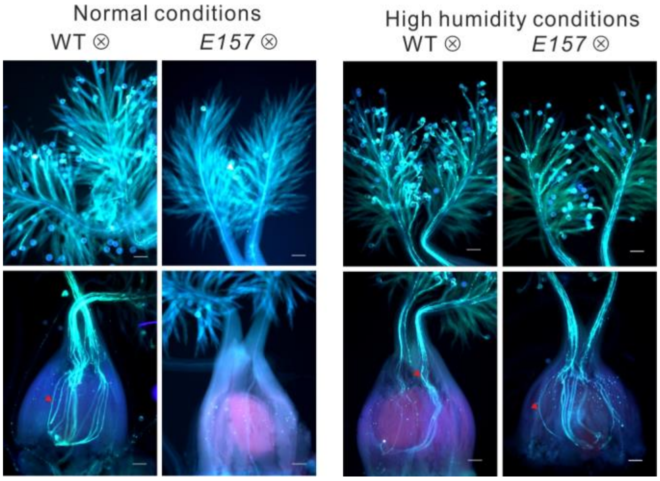

Supplementary Figure 7 | Pollen germination under different RH. *In vivo* pollen germination (upper panels) and pollen tube elongation (lower panels) of WT and *E157* pollen exposed to an RH of normal (30-60% RH), and high humidity (80-100% RH) conditions. Images were taken 1 h after pollination. Scale bar, 100 μm.

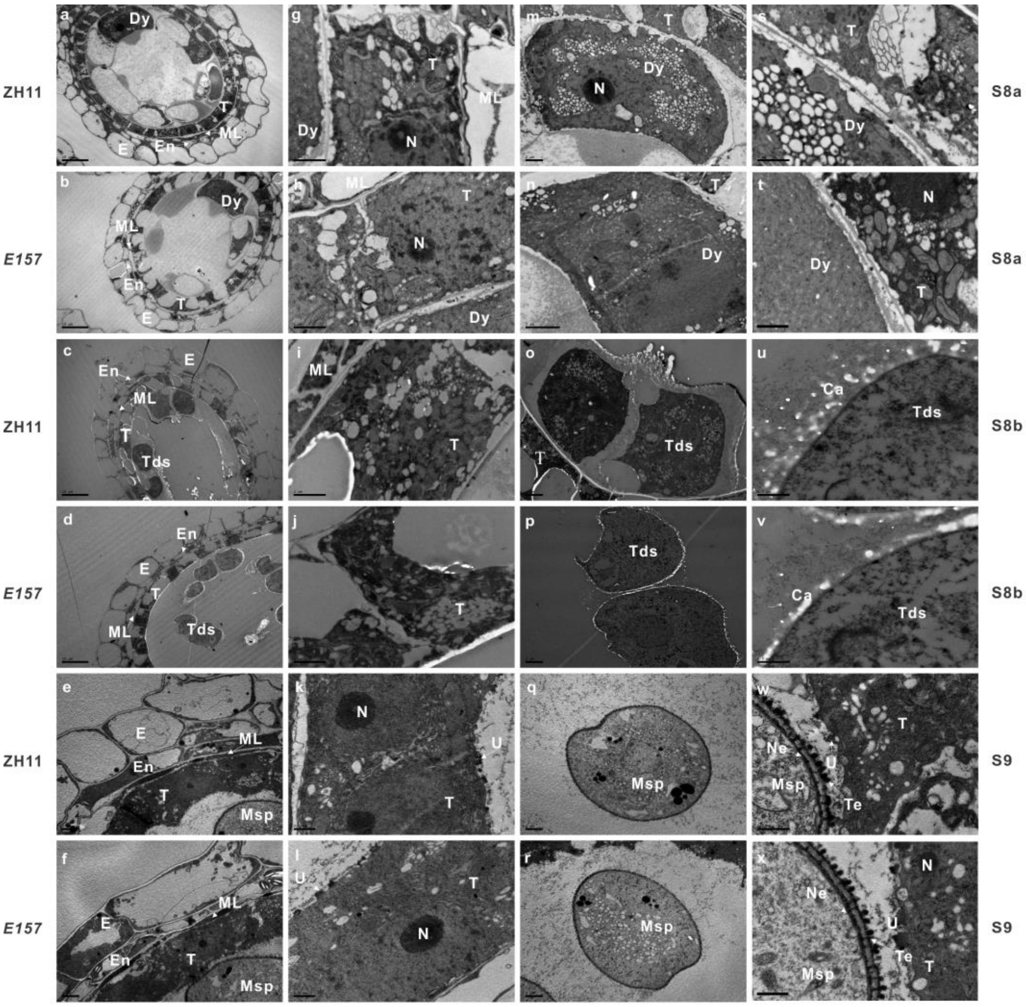

Supplementary Figure 8 | TEM analysis of the development of tapetum (a-l) and pollen wall (m-x) over the period S8-S9. Tapetum of wild-type ZH11: a, c, e, g, i, k; Tapetum of *E157* mutant: b, d, f, h, j, l; Pollen wall of wild-type ZH11: m, o, q, s, u, w; Pollen wall of *E157* mutant: n, p, r, t, v, x. Ca, callose wall; Dy, dyad; E, epidermis En, endothecium; ML, middle layer; Msp, microspore; N, nucleus; Ne, nexine; T, tapetum; Tds,Tetrads; Te, tectum; U, ubisch bodies. Scale bar for a-d, 10 μm, for e, f, m-r, 2 μm, for g-j, s, t, 1 μm, for k, l, u-x, 0.5 μm.

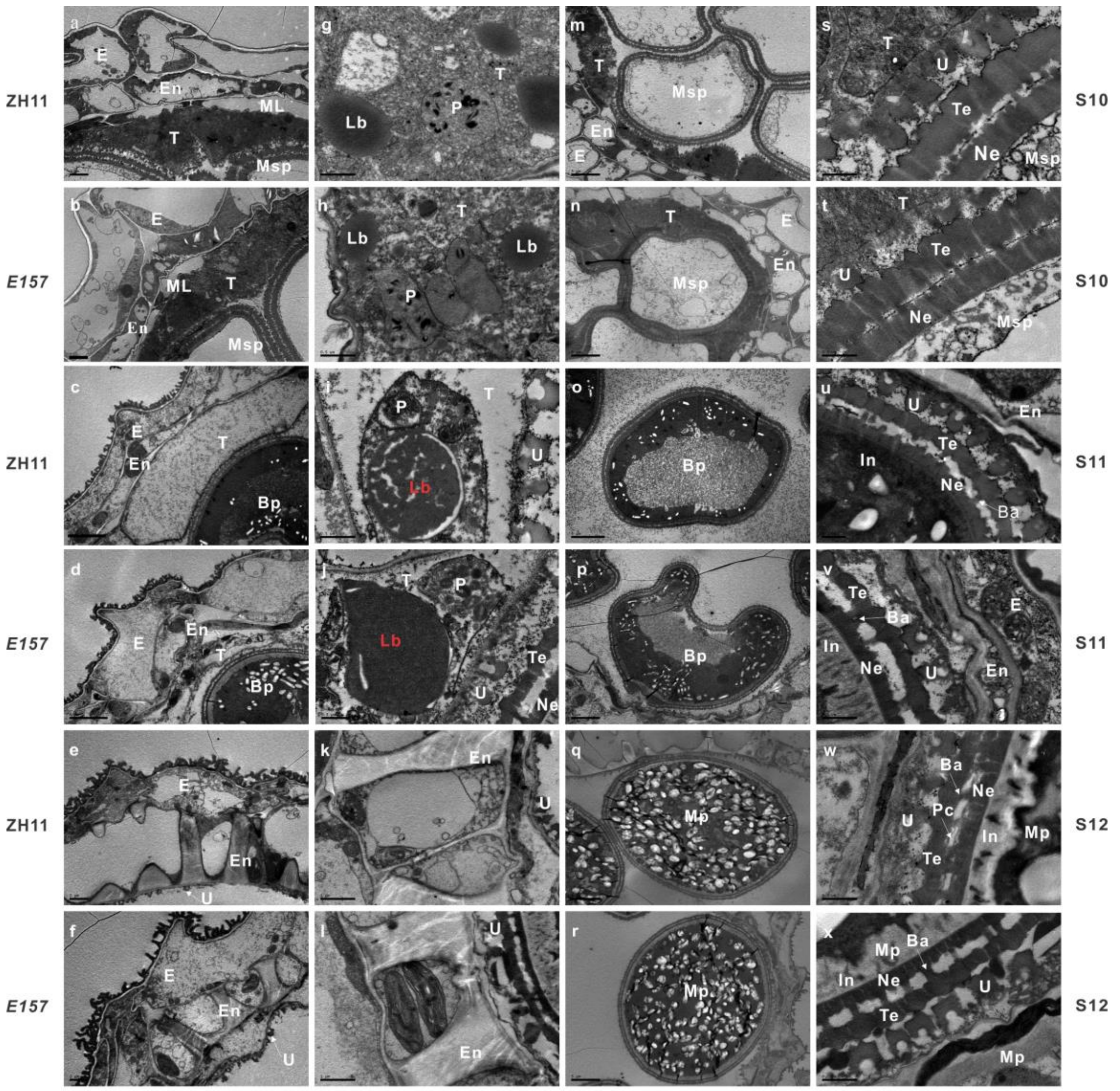

Supplementary Figure 9 | TEM analysis of the development of tapetum (a-l) and pollen wall (m-x) over the period S10-S12. Lipid body (Lb, in red font) is more intact in mutants *E157* than that in WT plants. The Tapetum of wild-type ZH11: a, c, e, g, i, k; Tapetum of *E157* mutant: b, d, f, h, j, l; Pollen wall of wild-type ZH11: m, o, q, s, u, w; Pollen wall of *E157* mutant: n, p, r, t, v, x. Ba, bacula; Bp, bicellular pollen; E, epidermis; En, endothecium; In, intine; Lb, lipid body; ML, middle layer; Msp, microspore; Mp, mature pollen; Ne, nexine; P, plastid; Pc, pollen coat; T, tapetum; Te, tectum; U, ubisch bodies. Scale bar for a, b, e, f, 2 µm, for c, d, m-r, 5 µm, for g-j, s-x, 0.5 µm, for k, l, 1 µm.

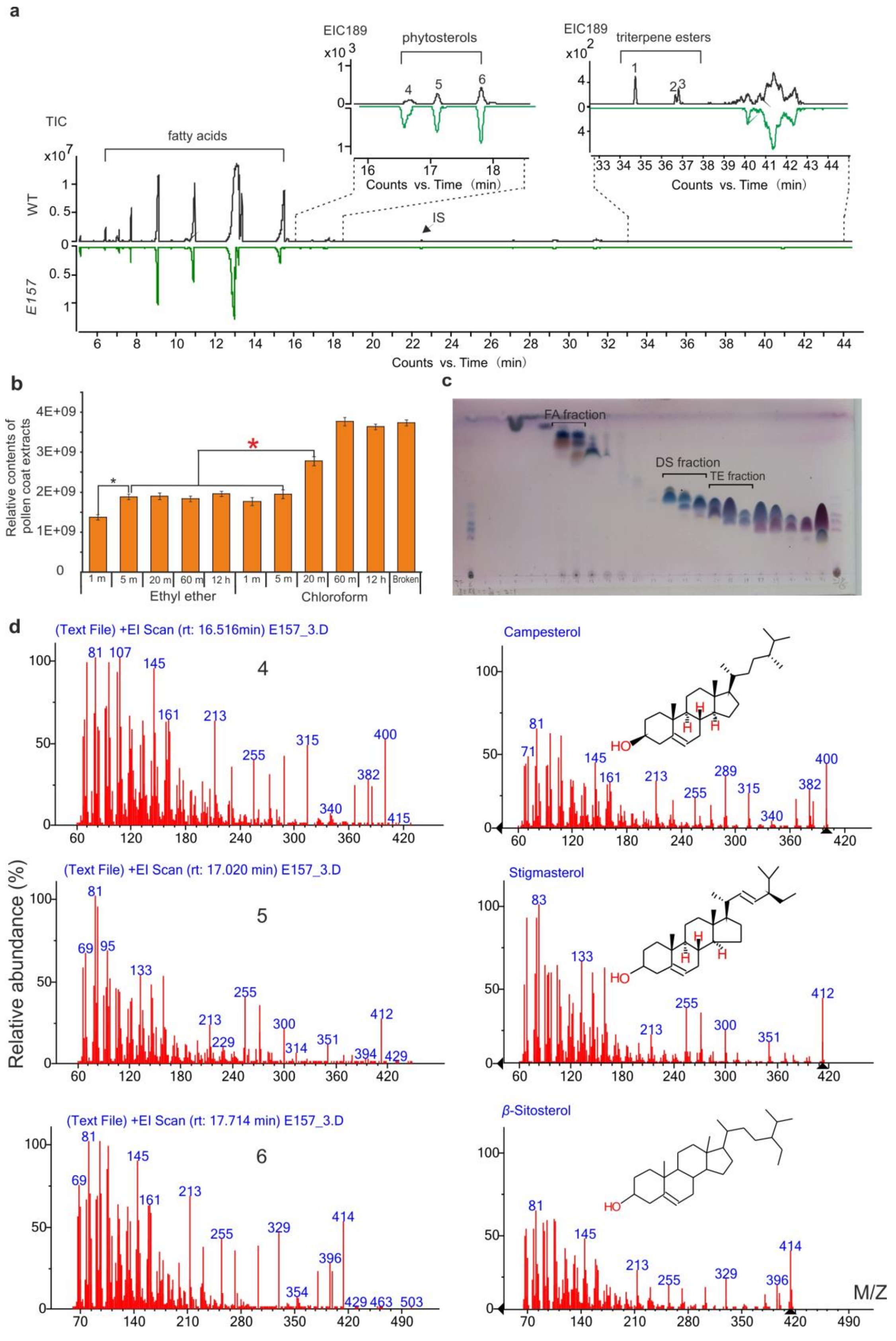

Supplementary Figure 10 | Method for pollen coat extraction and fractions of pollen coat extracts. (**a**) GC-MS profile of the ethyl ether extracts of pollen coat

from *E157* and WT plants. 1: poaceatapetol palmitic acid (16:0) ester; 2: poaceatapetol oleic acid (18:1) ester; 3: poaceatapetol stearic acid (18:0) ester; 4: campesterol; 5: stigmasterol; 6: β-sitosterol; IS: internal standard (betulin). Mass spectra of poaceatapetol esters were shown in Supplementary Figure 14. TIC, total ion chromatogram; EIC189, extracted ion chromatograms at m/z 189. **(b)** Amount of pollen coat extracts (PCE) by different solvents and extracting times. The amount was based on total GC-MS peak areas. The data are presented as means ± s.d., $n = 5$. The black asterisk indicates a significant difference ($P < 0.05$ by Student's *t*-test) between amounts of PCE for 1 min and 5 min ethyl ether extraction, indicating extraction of pollen coat material is incomplete by treatment in ethyl ether for 1 min. The red asterisk indicates significant different PCE amounts ($P < 0.05$ by Student's *t*-test) between both ethyl ether extractions (5 min to 12 h) and chloroform extractions for more than 20 min, also between 1 to 5 min and more than 20 min chloroform extractions. The PCE amounts from chloroform extractions more than 60 min do not differ from the extraction of broken pollen grains ($P > 0.05$ by Student's *t*-test). These results indicated that extractions by ethyl ether for more than 5 min or chloroform less than 5 minutes will specifically extract the pollen coat materials. **(c)** TLC analysis (hexane: dichloromethane = 2:1) of fractions obtained from silica column separations. About 200 g pollen grains were extracted by using ethyl ether for 20 min. The silica column was eluded by a gradient eluents system, and 20 g silica was used. FA fraction: fraction including fatty acids; DS fraction: fraction including dehydrated sterols; TE fraction: fraction including triterpene esters. **(d)** Mass spectra of three phytosterols from mutant *E157* (left) and commercial standards (right). 4: campesterol; 5: stigmasterol; 6: *β*-sitosterol.

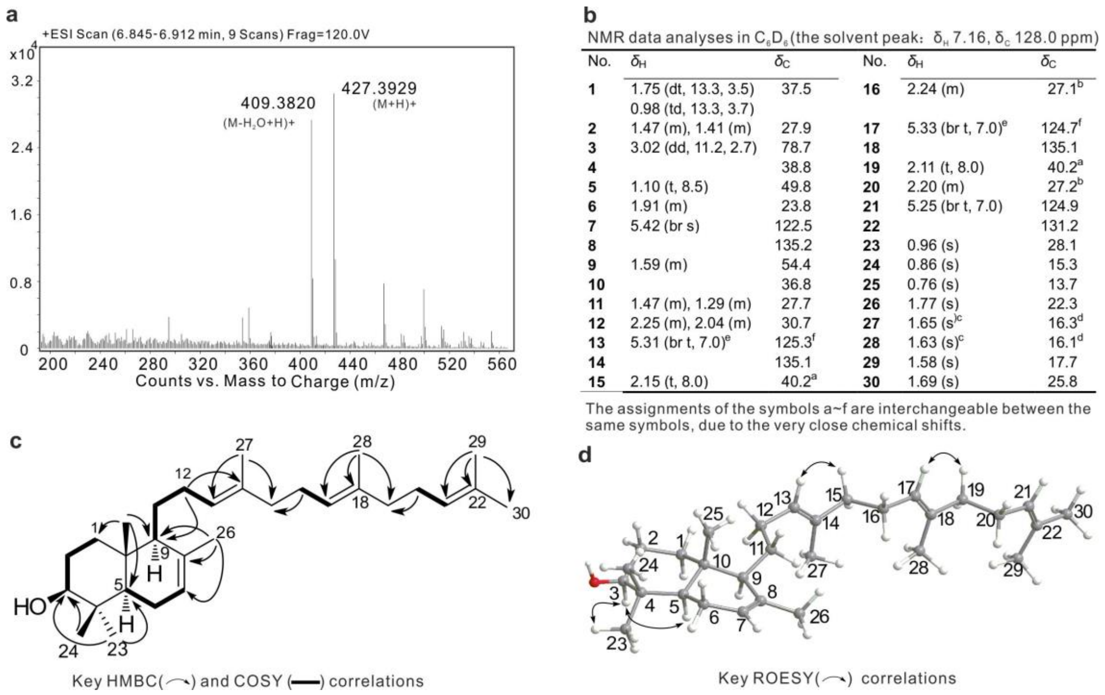

No.	δ_H	δ_C	No.	δ_H	δ_C
**1**	1.75 (dt, 13.3, 3.5)<br>0.98 (td, 13.3, 3.7)	37.5	**16**	2.24 (m)	27.1^b
**2**	1.47 (m), 1.41 (m)	27.9	**17**	5.33 (br t, 7.0)^e	124.7^f
**3**	3.02 (dd, 11.2, 2.7)	78.7	**18**		135.1
**4**		38.8	**19**	2.11 (t, 8.0)	40.2^a
**5**	1.10 (t, 8.5)	49.8	**20**	2.20 (m)	27.2^b
**6**	1.91 (m)	23.8	**21**	5.25 (br t, 7.0)	124.9
**7**	5.42 (br s)	122.5	**22**		131.2
**8**		135.2	**23**	0.96 (s)	28.1
**9**	1.59 (m)	54.4	**24**	0.86 (s)	15.3
**10**		36.8	**25**	0.76 (s)	13.7
**11**	1.47 (m), 1.29 (m)	27.7	**26**	1.77 (s)	22.3
**12**	2.25 (m), 2.04 (m)	30.7	**27**	1.65 (s)^c	16.3^d
**13**	5.31 (br t, 7.0)^e	125.3^f	**28**	1.63 (s)^c	16.1^d
**14**		135.1	**29**	1.58 (s)	17.7
**15**	2.15 (t, 8.0)	40.2^a	**30**	1.69 (s)	25.8

Supplementary Figure 11 | The structural identification of OsOSC12 product. (**a**) Mass spectrum of OsOSC12 product as derived from Agilent 6540 Quadrupole time-of-flight mass spectrometry. The positive electrospray ionization (ESI) mode was used. (**b**) The assignments of ^{1}H and ^{13}C NMR data of OsOSC12 product, poaceatapetol (polypoda-7,13*E*,17*E*,21-tetraene-3β-ol). The full assignments and connectivity were determined by ^{1}H–^{1}H COSY, ROESY, HSQC, and HMBC spectra (Supplementary Data 1). (**c**) Key correlations of HMBC and COSY. (d) Key corrections of ROESY.

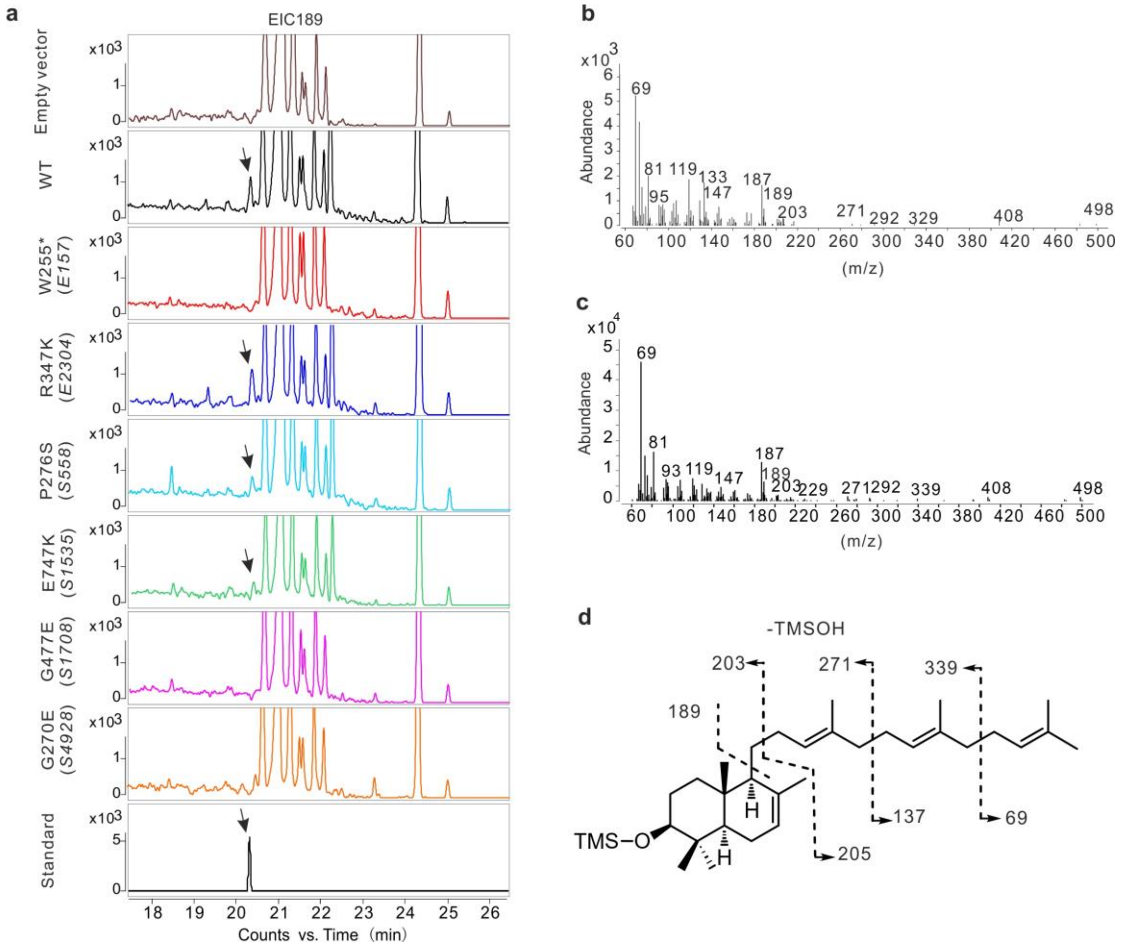

Supplementary Figure 12 | Functional analysis of OsOSC12 in *Pichia pastoris*. (**a**) GC-MS profiles of extracts from *Pichia pastoris* strains heterologously expressing wild-type and mutants' OsOSC12 proteins. The WT protein was deduced from synthetic gene *SnOsOSC12* (Supplementary Data 3) with yeast preferred codon usage. All mutants were generated by PCR based site-directed mutagenesis. The arrow indicated the peak of OsOSC12 product. Standard, purified poaceatapetol; EIC189, extracted ion chromatograms at *m/z* 189. (**b**) Mass spectrum of trimethylsilyl derivatives of the OsOSC12 product extracted from *Pichia pastoris*. (**c**) Mass spectrum of poaceatapetol standard purified from rice panicles. (**d**) The proposed fragmentation of trimethylsilyl (TMS) derivative of poaceatapetol.

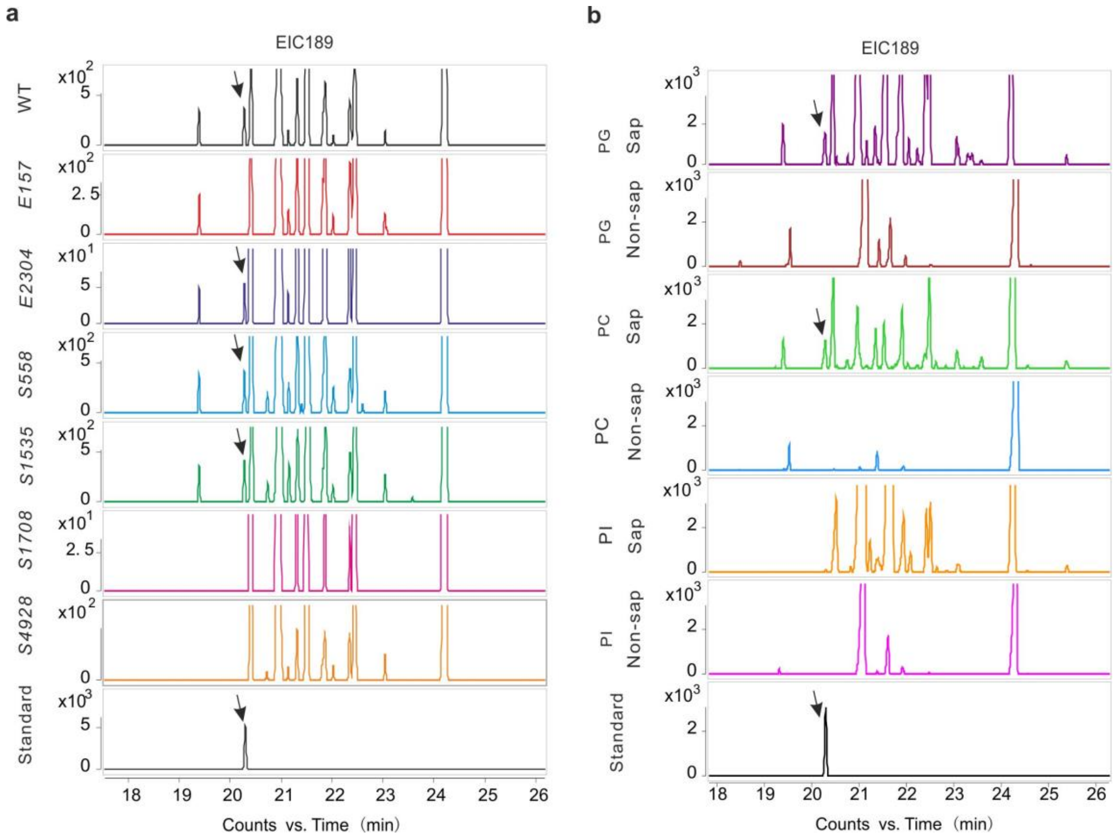

Supplementary Figure 13 | Identification of poaceatapetol derivatives in pollen coat. (**a**) GC-MS profiles of the triterpene alcohols of pollen extractions from *OsOSC12* mutants and WT plants. (**b**) GC-MS profiles of the triterpene alcohols from extracts of non-saponified (Non-sap) and saponified (Sap) pollen grains (PG), pollen coat (PC), and pollen inside (PI) of WT plants. The arrow indicates the poaceatapetol peak. Standard, purified poaceatapetol; EIC189, extracted ion chromatograms at *m/z* 189. Poaceatapetol was identified in the saponified WT pollen grains and pollen coat materials, but it is not detected in the non-saponified samples of the WT pollen grains and pollen inside materials. Saponification treatment generates free alcohols and triterpenes from modified triterpenoids.

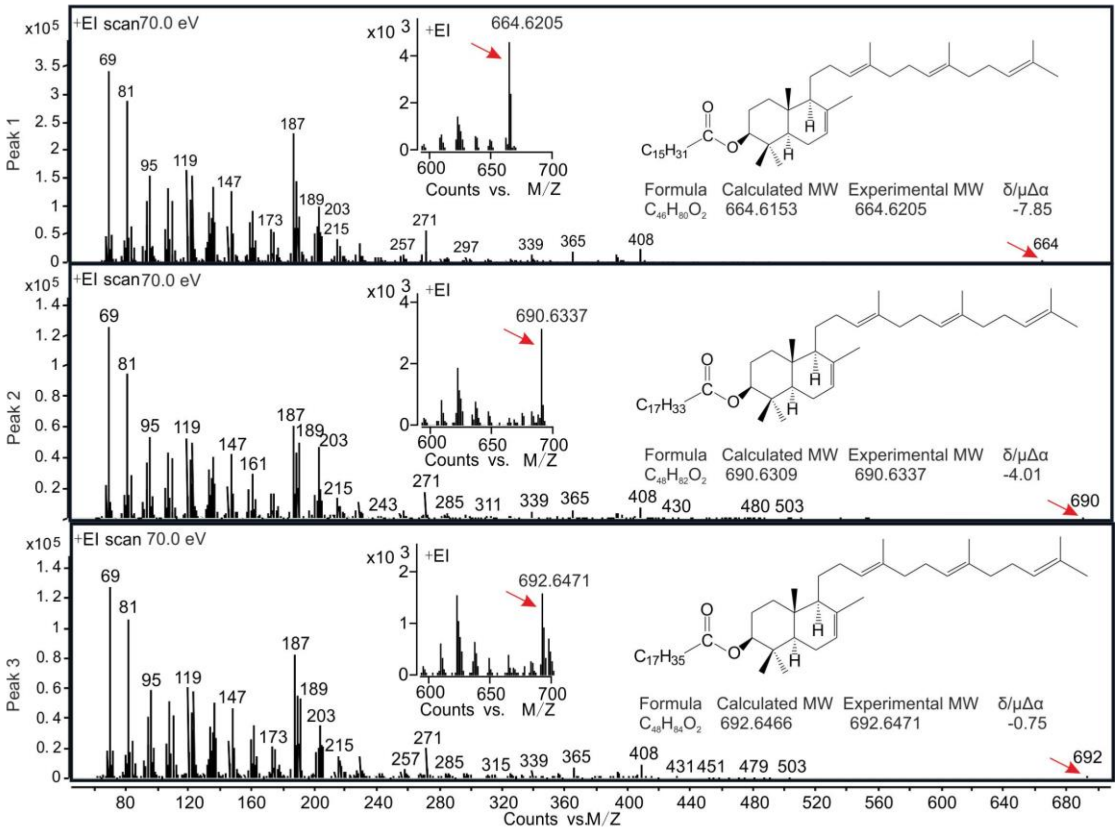

Supplementary Figure 14 | The mass spectra and predicted structures of poaceatapetol fatty acid esters. GC-MS analysis revealed three peaks 1, 2, and 3 (the region of triterpene esters in Supplementary Fig. 10) from the WT pollen coat sample. The mass spectrum of each poaceatapetol fatty acid ester is zoomed in to show its molecular ion (see red arrows). Samples were analyzed by using Agilent 7200A GC/Q-TOF with an electron ionization of 70 eV. High resolution mass analysis suggested that these three peaks are C16:0, C18:1 and C18:0 fatty acid esters of poaceatapetol.

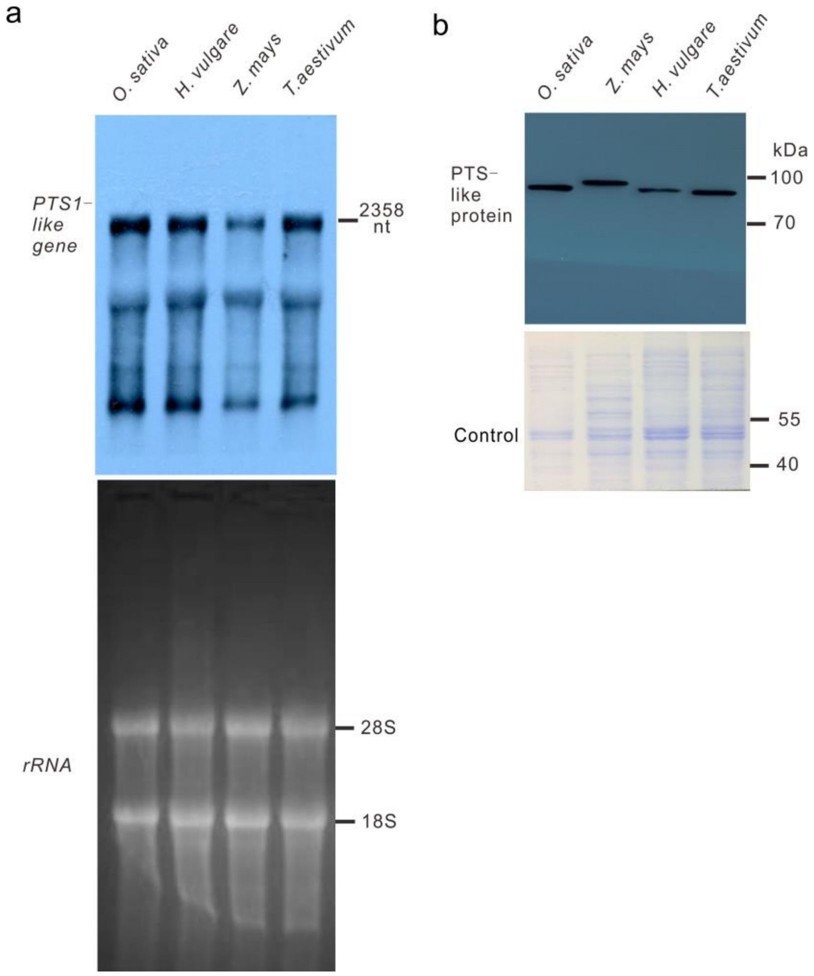

Supplementary Figure 15 | Transcription and expression of *OsOSC12*-like genes. (**a**) Northern blotting analysis of transcription in the anthers of four major cereal species. (**b**) Western blotting demonstrates the presence of OsOSC12-like proteins in the anthers of other cereal species. The negative controls for Northern and Western blotting experiments were, respectively, 18S and 28S rRNA, and SDS-PAGE gel stained by coomassie brilliant blue.

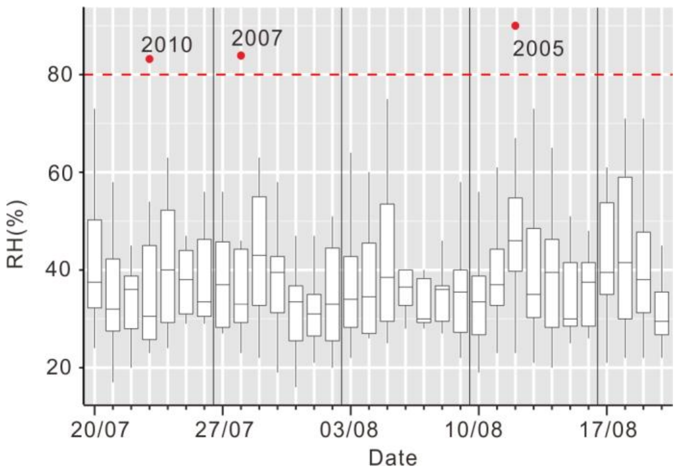

Supplementary Figure 16 | Box plot for the day average RH around rice anthesis period (July 20th to August 17th) in Urumqi city, Xinjiang China (from 2005-2014). Data were provided by the Xinjiang Uygur Autonomous Region Meteorological Information Centre.

Supplementary Table 1. EMS* and SAZ† induced mutations in *OsOSC12*.

Mutant	Mutation event	Position	Predicted amino acid change	Population
E248	A -> G	Intron 9	-	EMS
S1708	G -> A	Exon 10	G^{1430} -> E	SAZ
E572	A -> G	Intron9	-	EMS
S4312	C -> T	Intron17	-	SAZ
S1535	G -> A	Exon18	E^{2239} -> K	SAZ
S558	C -> T	Exon6	P^{826} -> S	SAZ
S134	G -> A	Intron6	-	SAZ
S4888	G -> A	Intron6	-	SAZ
E2304	G -> A	Exon7	R^{1031} -> K	EMS
E157	G -> A	Exon6	W^{764} -> stop	EMS
E997	A -> G	Intron6	-	EMS
E1501	C -> T	Exon7	L^{961} -> L	EMS
S4175	G -> A	Intron6	-	SAZ
S4928	G -> A	Exon6	G^{809} -> E	SAZ

*EMS and †SAZ represent the ethylmethylsulfone and sodium azide.

Supplementary Table 2. **Genetics analysis of *OsOSC12* mutants.**

BC_3F_2	Total	Fertility	Sterility	Ratio	χ^2 (3:1)	*P* value
E157♀× WT♂	366	276	90	3.07:1	0.033	>0.05
S1708♀× WT♂	172	132	40	3.3:1	0.279	>0.05
S4928♀× WT♂	190	143	47	3.04:1	0.007	>0.05

Note: F_1 hybrids from crosses between the three male sterile mutants (*E157*, *S1708* and *S4928*) and the WT appeared morphologically normal and were fully self-fertile. The F_2 progeny of each cross segregated consistently in the ratio of three fertile to one sterile.

Supplementary Table 3. **Sequences of oligonucleotide primers used.**

Name	Sequence (5'-3')
OSCA8_1	TCCCAGGCCCACTGTTTACC
OSCA8_2	GCCATCTTCGCCAACCCAT
OSACT_1	TCCATCTTGGCATCTCTCAG
OSACT_2	GTACCCGCATCAGGCATCTG
EX6-8_1	GAGGTCAAGTCGTCTTCTGCAATTA
EX6-8_2	ATTTGTCTGCGCTCTGCACATG
EX8-10_1	GCTTAAAGGTAAATTTCAGGCTTCC
EX8-10_2	CGATCAGAATCAATTAAACCCAGAC
EX16-18_1	TCATCCTTAGATTAATTAGCCGACA
EX16-18_2	CATAAGGATCTCATAAAATCGACCA
OSC8S	ATGTGGAAGCTCAAGATTGCCGAG
OSC8A	TCAAGTTGGCGCTGTTGTACTTGC
ANTISENSE_1	TCCCAGGCCCACTGTTTACC
ANTISENSE_2	TAATACGACTCACTATAGGGGGTCATGTATCCATTTTCTTGC
SENSE_1	TAATACGACTCACTATAGGGTCCCAGGCCCACTGTTTACC
SENSE_2	GGTCATGTATCCATTTTCTTGC
E157-Mu_1	CAACCAGGACGACTTTAGAGTCACTTCCGAATG
E157-Mu_2	CATTCGGAAGTGACTCTAAAGTCGTCCTGGTTG
E2304-Mu_1	ATTAGTTACATGAGAAAAAAGGCTCTATACCAAATTGCTG
E2304-Mu_2	CAGCAATTTGGTATAGAGCCTTTTTTCTCATGTAACTAAT
S558-Mu_1	GGTAAAAAGTTTGTCGGTTCTATTACAAGATTGGTTA
S558-Mu_2	TAACCAATCTTGTAATAGAACCGACAAACTTTTTACC
S1535-Mu_1	CCCTATATGGGCTCTTGGAAAGTACCAGAAATTAGTATT
S1535-Mu_2	AATACTAATTTCTGGTACTTTCCAAGAGCCCATATAGGG
S1708-Mu_1	TCAGGTTGCCGATCAAGAATGGCAGGTTTCAGATTG
S1708-Mu_2	CAATCTGAAACCTGCCATTCTTGATCGGCAACCTGA
S4928-Mu_1	CCATGTCTTACTTGTATGAGAAAAAGTTTGTCGGTCCT
S4928-Mu_2	AGGACCGACAAACTTTTTCTCATACAAGTAAGACATGG

The bases with underline are the mutation sites for each mutant.

Supplementary Table 4. **The treatments in exogenous application experiments.**

Abbreviated names	The components and concentration of solution* for exogenous application treatments
E157-mock	-
WT-mock	-
1/4 WT	1/4 dilution PCE from 5g WT pollen grains (50 mg mL^{-1})
1/5 WT	1/5 dilution PCE from 5g WT pollen grains (40 mg mL^{-1})
3/20 WT	3/20 dilution PCE from 5g WT pollen grains (30 mg mL^{-1})
1/10 WT	1/10 dilution PCE from 5g WT pollen grains (20 mg mL^{-1})
1/20 WT	1/20 dilution PCE from 5g WT pollen grains (10 mg mL^{-1})
1/4 E157	1/4 dilution PCE from 5g *E157* pollen grains (25 mg mL^{-1})
1/5 E157	1/5 dilution PCE from 5g *E157* pollen grains (20 mg mL^{-1})
3/20 E157	3/20 dilution PCE from 5g *E157* pollen grains (15 mg mL^{-1})
1/10 E157	1/10 dilution PCE from 5g *E157* pollen grains (10 mg mL^{-1})
1/20 E157	1/20 dilution PCE from 5g *E157* pollen grains (5 mg mL^{-1})
FA fraction	fatty acid fractions from WT PCE (20 mg mL^{-1})
DS fraction	dehydrated sterol fractions from WT PCE (20 mg mL^{-1})
TE fraction	triterpene ester fractions from WT PCE (20 mg mL^{-1})
16:0+18:0+18:3	16:0 18:0 and 18:3 with ratio of 23:19:49 (20 mg $mL^{-1\dagger}$)
16:0+18:3	16:0 and 18:3 with ratio of 23:49 (20 mg $mL^{-1\dagger}$)
18:0+18:3	18:0 and 18:3 with ratio of 19:49 (20 mg $mL^{-1\dagger}$)
16:0+18:0	16:0 and 18:0 with ratio of 23:19 (20 mg $mL^{-1\dagger}$)
16:0	palmitic acid (20 mg mL^{-1})
18:3	linolenic acid (20 mg mL^{-1})
18:0	stearic acid (20 mg mL^{-1})

* All the fractions and chemicals were dissolved in hexane. † represents total concentration.
