# Supplementary material for: Deficiency of a triterpene pathway results in humidity-sensitive genic male sterility in rice
Source: Nat Commun. 2018 Feb 9;9:604. doi: 10.1038/s41467-018-03048-8 (PMC5807508; doi:10.1038/s41467-018-03048-8)
Supplement: Supplementary file 3 — Description of Additional Supplementary Files [file 41467_2018_3048_MOESM3_ESM.pdf]

**Description of Supplementary Files**

File Name: Supplementary Data 1
Description: NMR data of polypoda-7,13E,17E,21-tetraene-3-beta-ol.

File Name: Supplementary Data 2
Description: Physicochemical data of polypoda-7,13E,17E,21-tetraene-3-beta-ol.

File Name: Supplementary Data 3
Description: The synthetic OsOSC12 sequence used for heterologous expression in *Pichia pastoris*.

File Name: Supplementary Movie 1
Description: The time lapse sequence showing pollen adhering onto the WT and E157 stigmas. The time elapsed between consecutive images was 2 s, and the whole period covered was 10.7 min. The resulting video is presented at a rate of ten frames per second. Scale bar, 50 µm.
