# Supplementary material for: Deficiency of a triterpene pathway results in humidity-sensitive genic male sterility in rice
Source: Nat Commun. 2018 Feb 9;9:604. doi: 10.1038/s41467-018-03048-8 (PMC5807508; doi:10.1038/s41467-018-03048-8)

NMR data of polypoda-7,13*E*,17*E*,21-tetraene-3-beta-ol

Content list:

S1. ^{1}H NMR spectrum (800 MHz, C_6D_6)

S2. ^{13}C NMR spectrum (200 MHz, C_6D_6)

S3. Enlarged ^{13}C NMR spectrum (200 MHz, C_6D_6)

S4. HSQC spectrum (800 MHz, C_6D_6)

S5. ^{1}H-^{1}H COSY spectrum (800 MHz, C_6D_6)

S6. ROESY spectrum of (800MHz, C_6D_6)

S7. HMBC spectrum (800 MHz, C_6D_6)

S8. Enlarged HMBC spectrum (800 MHz, C_6D_6)

S1. ^{1}H NMR spectrum (800 MHz, C_6D_6)

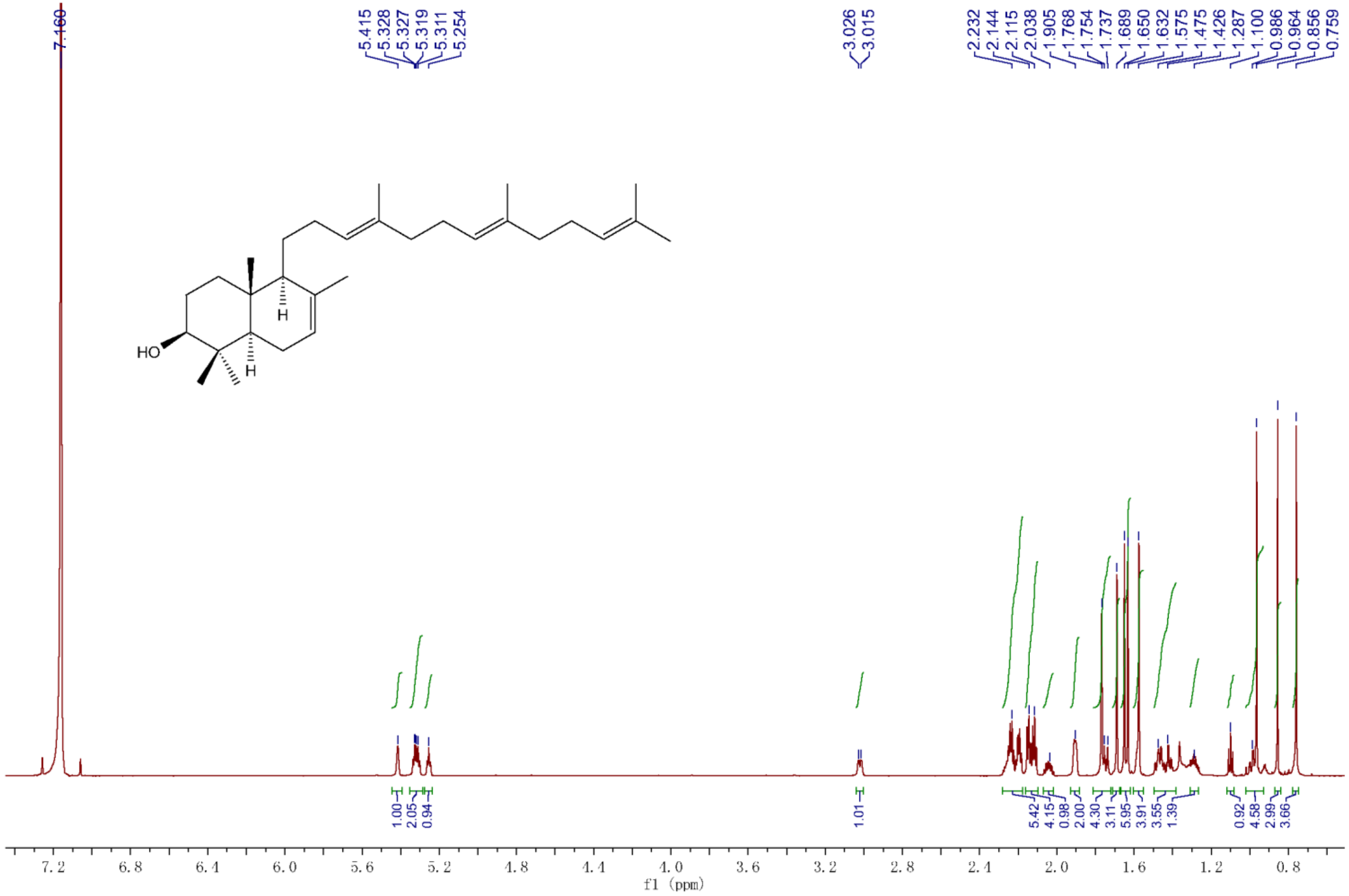

S2. ^{13}C NMR spectrum (200 MHz, C_6D_6)

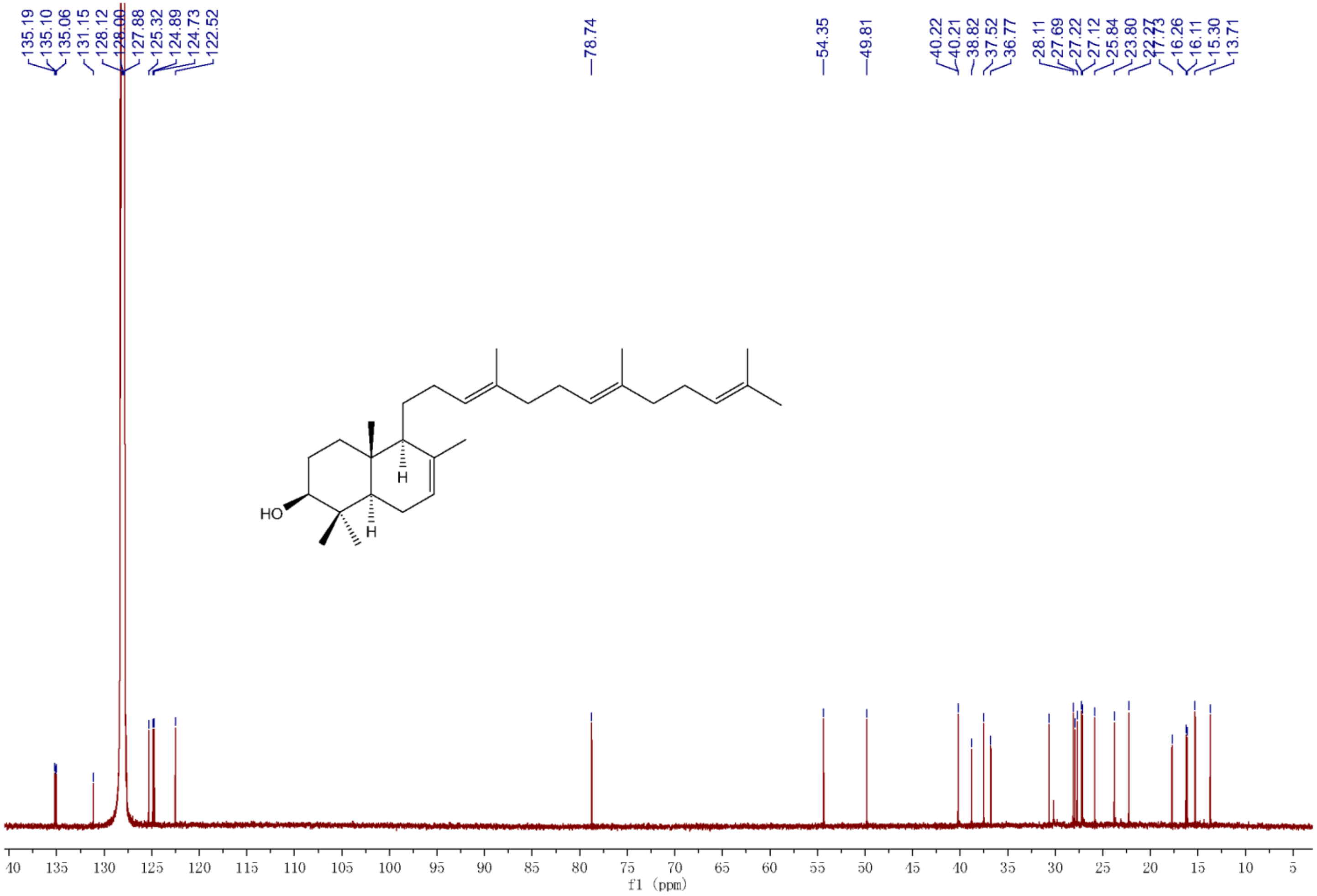

S3. Enlarged ^{13}C NMR spectrum (200 MHz, C_6D_6)

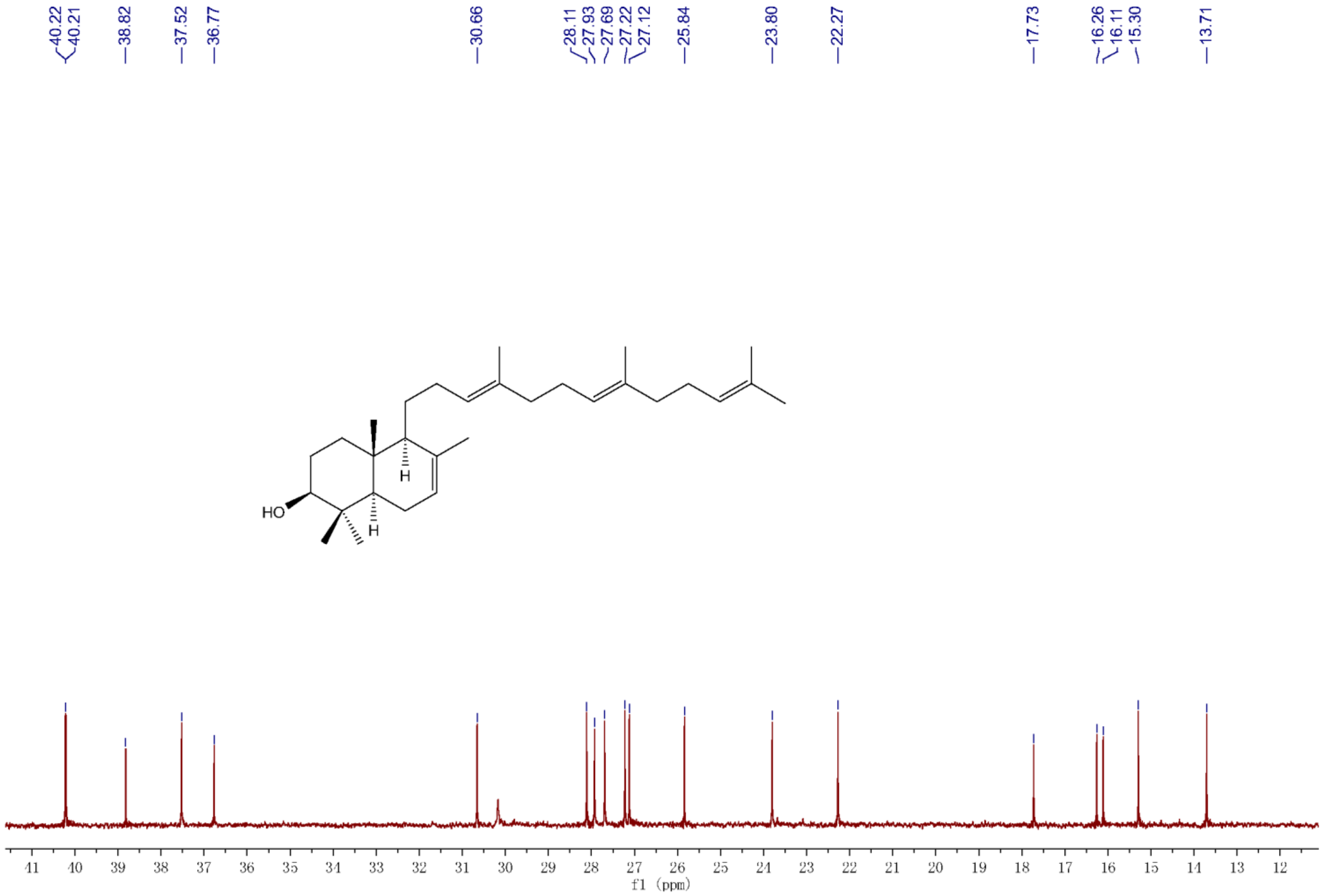

S4. HSQC spectrum (800 MHz, C_6D_6)

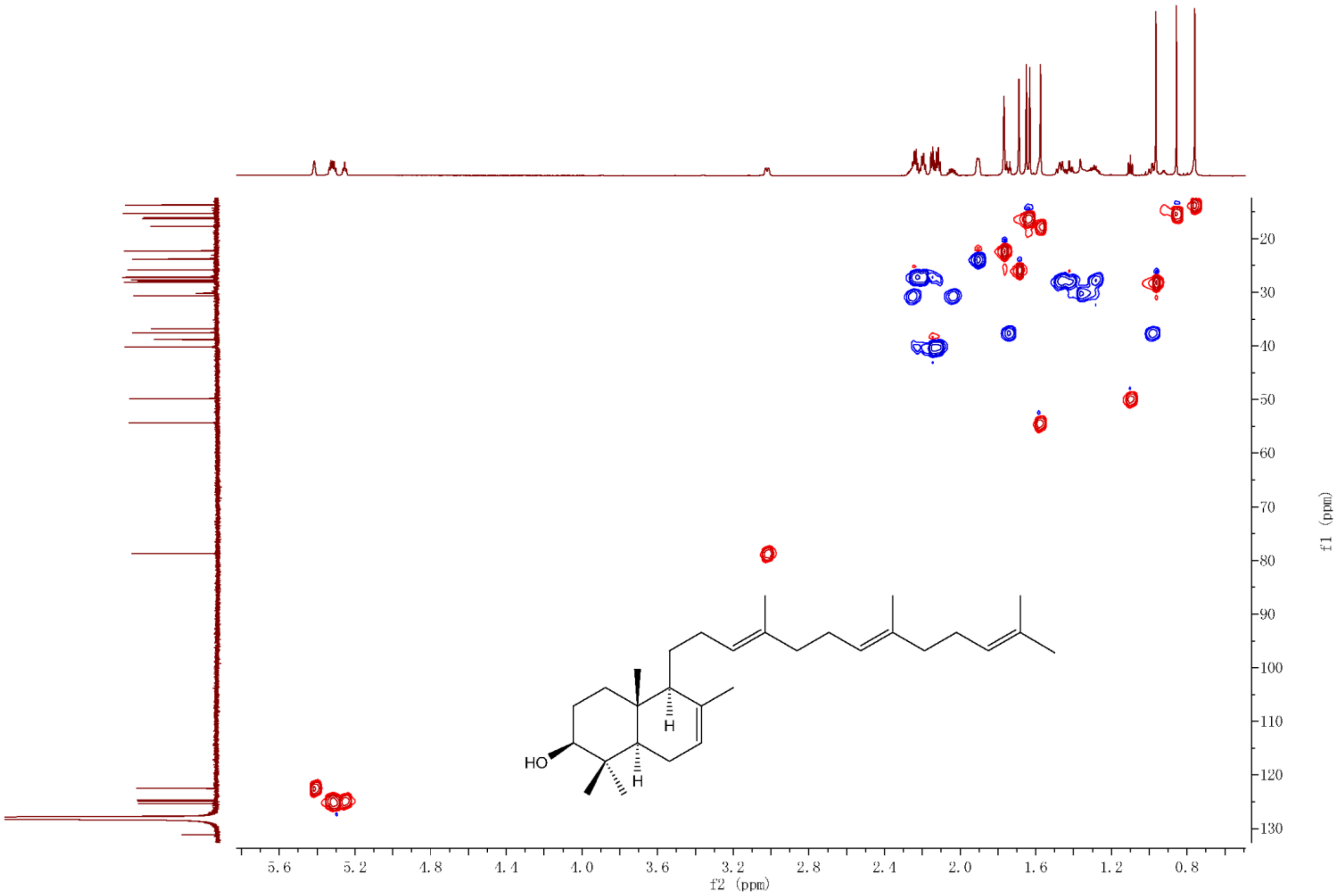

S5. ^{1}H-^{1}H COSY spectrum (800 MHz, C_6D_6)

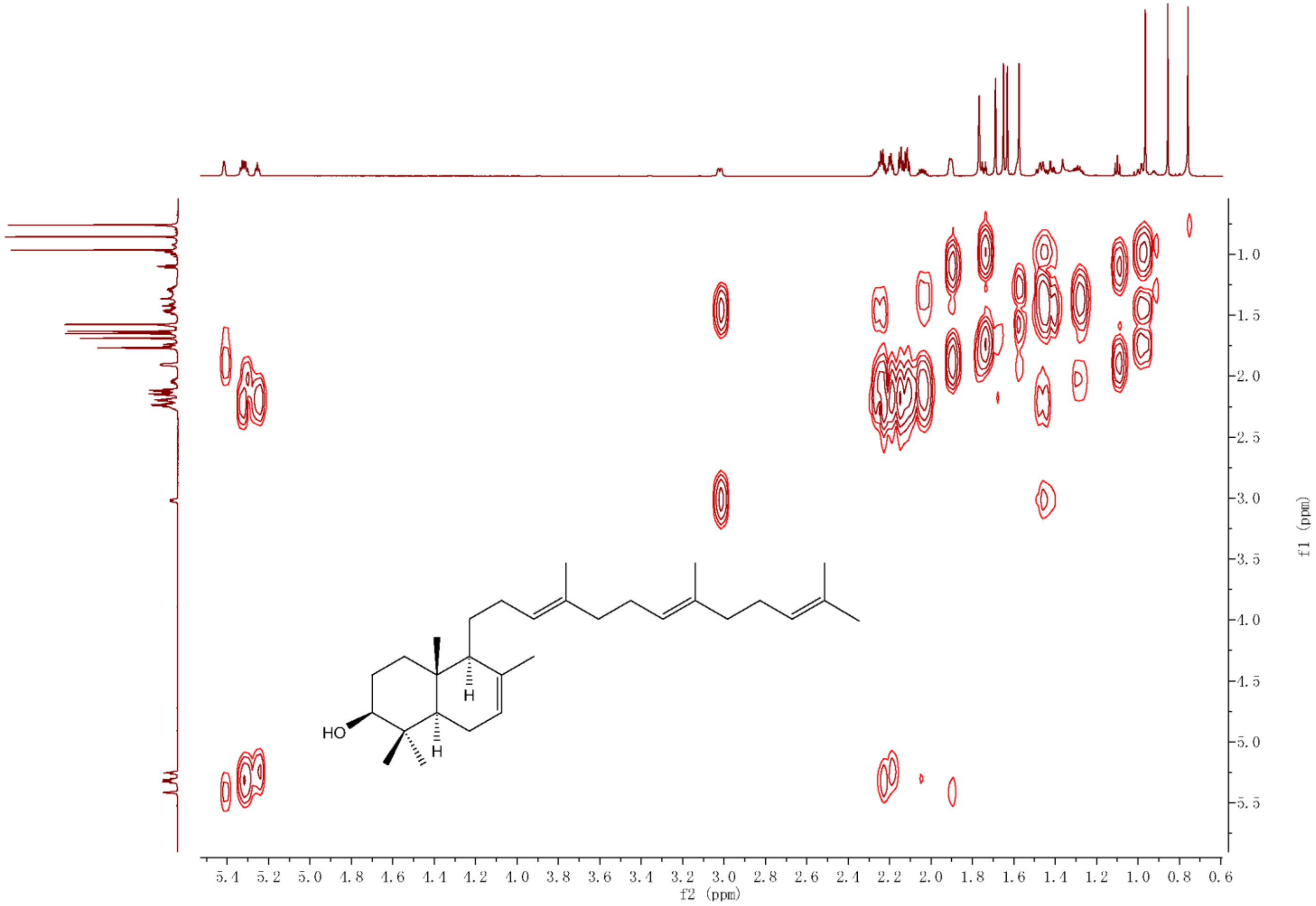

S6. ROESY spectrum of (800MHz, C_6D_6)

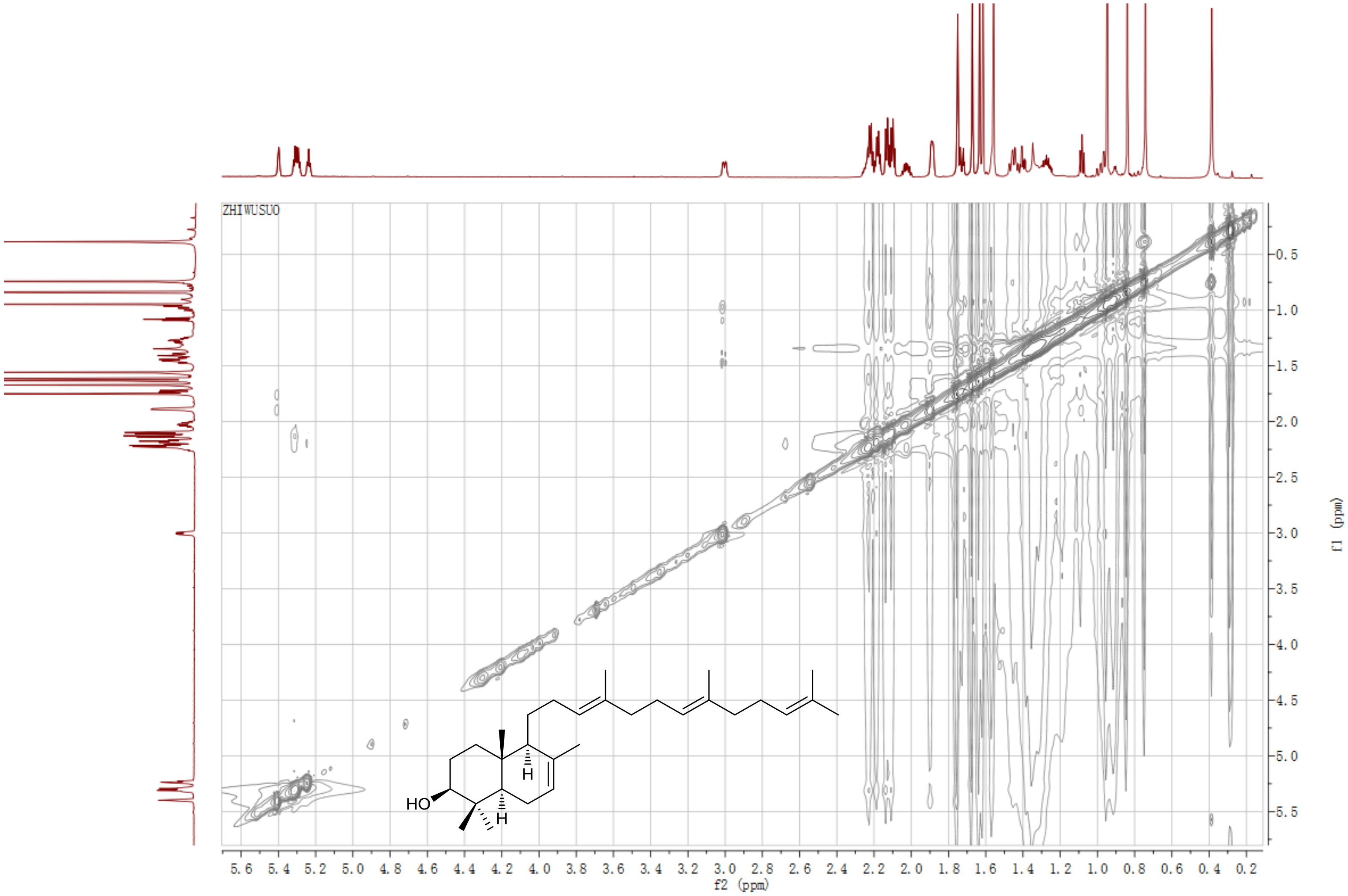

S7. HMBC spectrum (800 MHz, C_6D_6)

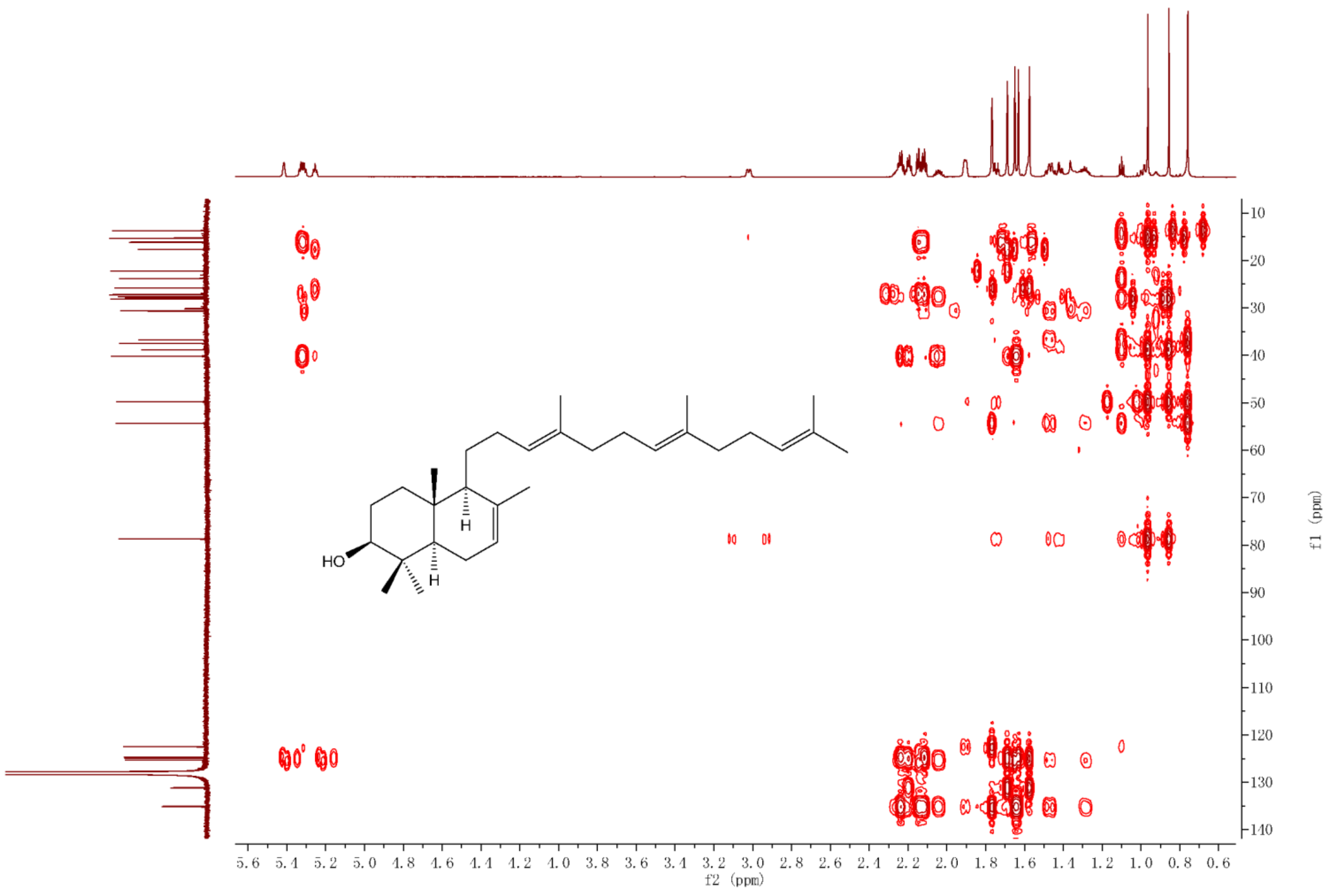

S8. Enlarged HMBC spectrum (800 MHz, C_6D_6)

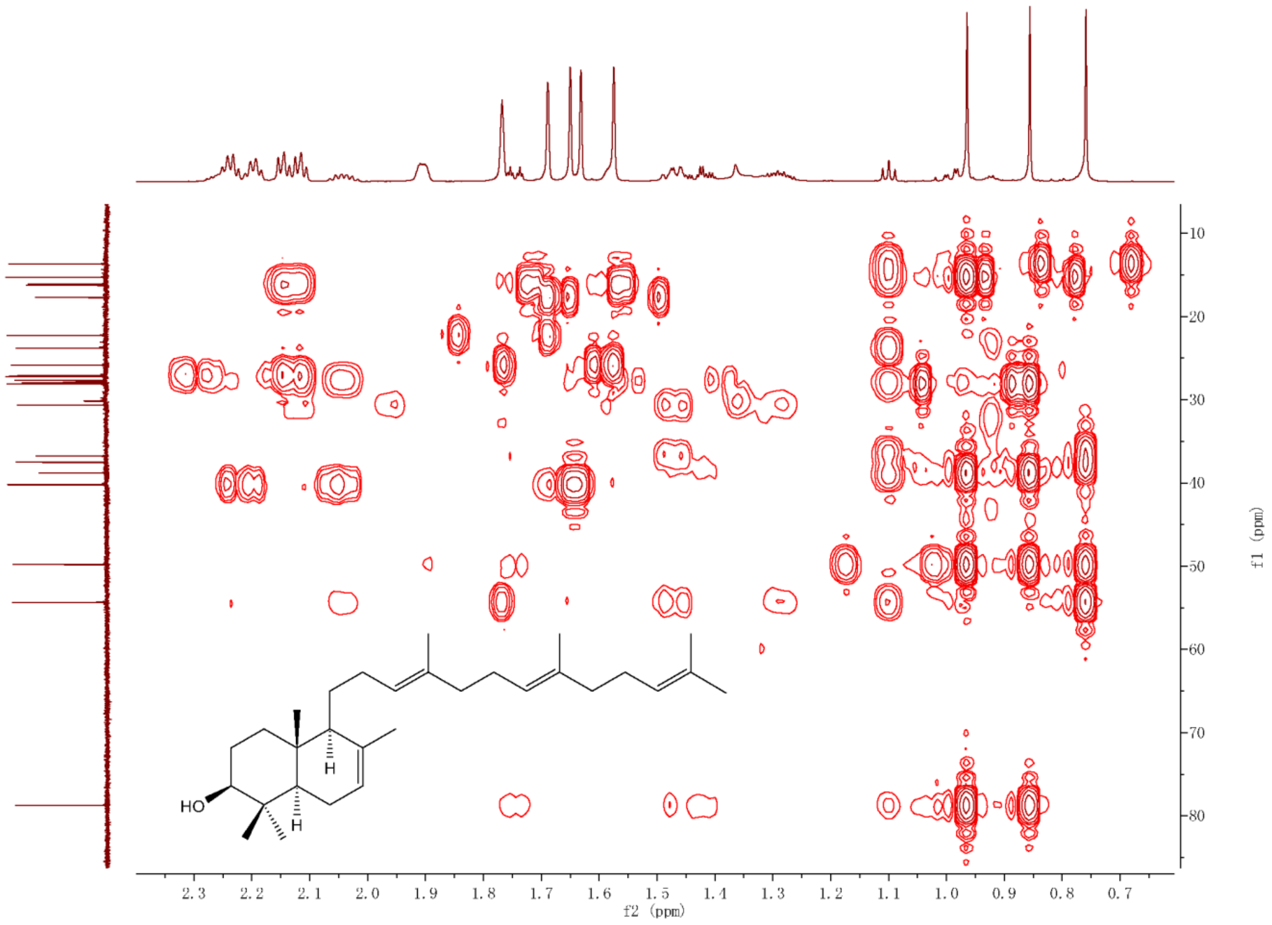

Supplement: Supplementary file 4 — Supplementary Data 1 [file 41467_2018_3048_MOESM4_ESM.pdf]
