# Supplementary material for: Deficiency of a triterpene pathway results in humidity-sensitive genic male sterility in rice
Source: Nat Commun. 2018 Feb 9;9:604. doi: 10.1038/s41467-018-03048-8 (PMC5807508; doi:10.1038/s41467-018-03048-8)

Physicochemical data of polypoda-7,13*E*,17*E*,21-tetraene-3-beta-ol

S1. Infrared spectroscopy.
S2. UV spectroscopy (dissolved in CH_3OH).

S1. IR spectroscopy

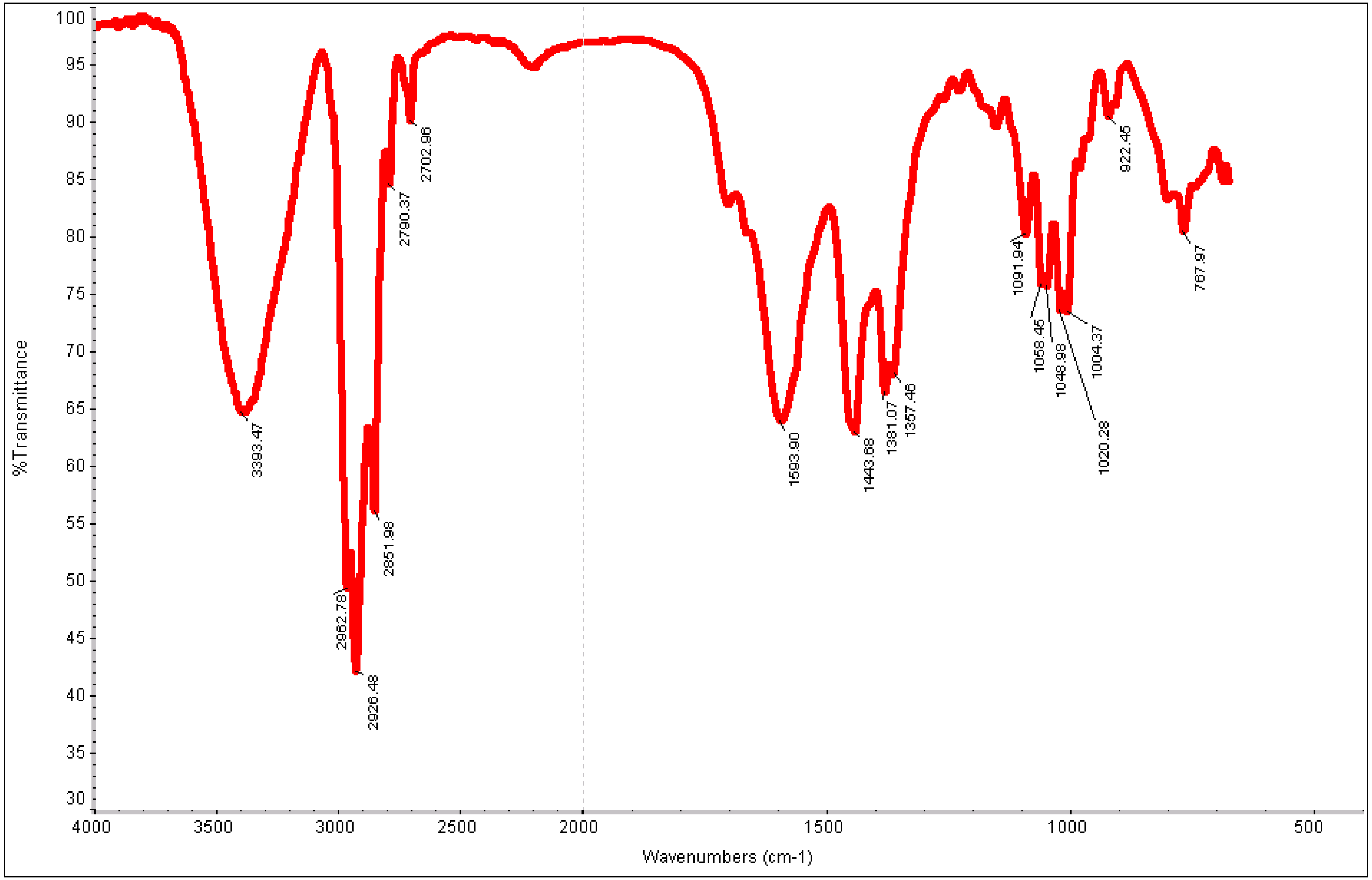

S2. UV spectroscopy (dissolved in CH_3OH).

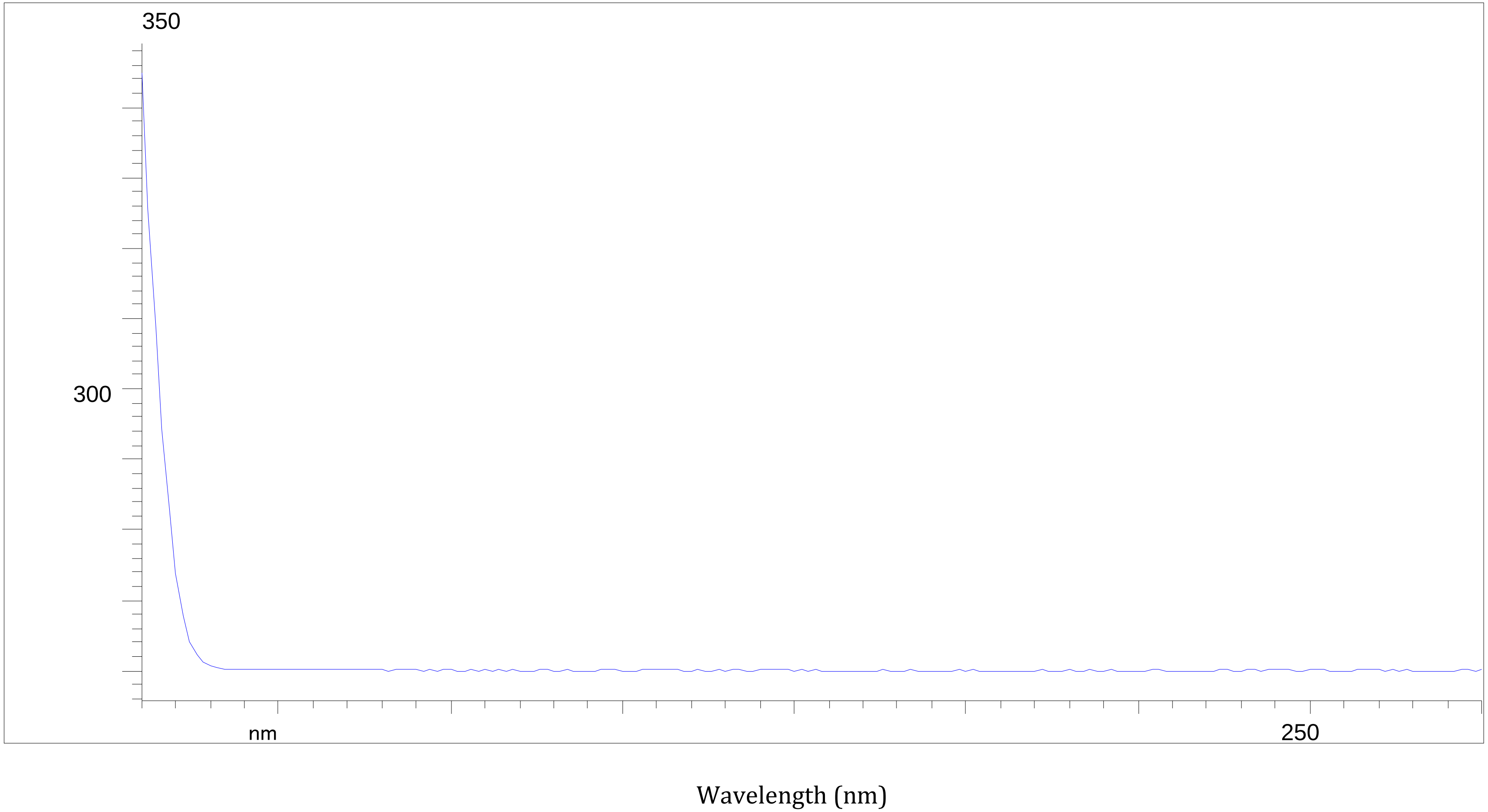

Supplement: Supplementary file 5 — Supplementary Data 2 [file 41467_2018_3048_MOESM5_ESM.pdf]
